# An Unusual Colocynth Symptom

**Published:** 1887-04

**Authors:** R. Gibson Miller

**Affiliations:** Glasgow, Scotland


### AN UNUSUAL COLOCYNTH SYMPTOM.

R. Gibson Miller, M. D., Glasgow, Scotland.

David ———, aged thirty-nine, packing-box maker, has suffered constantly for seven years from the following symptoms:

Griping pain in the stomach, extending through to the back, so severe as to cause him to moan and toss about. The pain begins at eleven A. M.; lasts till about twelve P. M.

The pain forces him to bend double, and is aggravated by straightening himself out or by touch, but ameliorated by pressure, especially on a sharp corner, by drawing up the legs *and during and two hours after eating.*

Another branch of the pain extends from the stomach around the left hypochondrium. The pain in the back is ameliorated by hard pressure or stretching out.

Sensation as if the food had stuck in supra-sternal fossa.

Rumbling in the stomach in moving. Yellow, liquid, painless stools, with great urging and tenesmus before and during, but complete relief after stool.

Colocynth $^{1m}$ (Heath), was given, and a sharp aggravation for ten days was the result. The Coloc. was stopped, and Sac. lac. given, and he immediately began to improve, the symptoms disappearing in reverse order to which they came, so that within six weeks he was absolutely free from pain, and has continued well ever since.

In giving the Colocynth I was not satisfied I had the right remedy, but being unable to find any nearer to the symptoms gave it.

The relief from eating was very marked, and is so opposed to all the usual symptoms of the drug that it made me hesitate about giving it. In Allen, Symptom 323, which reads : "Beer (after eleven A. M.) caused violent griping pains in the stomach, coming on in paroxysms, and only disappearing *after dinner*," is the nearest I can find, as it has both the time and relief after eating, although in this case it was not due to beer, and I should be glad to know if any one else has noticed this relief after eating under Colocynth.

